# Tablet Formulations of Polymeric Electrospun Fibers for the Controlled Release of Drugs with pH-Dependent Solubility

**DOI:** 10.3390/polym14102127

**Published:** 2022-05-23

**Authors:** Valeria Friuli, Silvia Pisani, Bice Conti, Giovanna Bruni, Lauretta Maggi

**Affiliations:** 1 Department of Drug Sciences, University of Pavia, Via Taramelli 12, 27100 Pavia, Italy; valeria.friuli@unipv.it (V.F.); bice.conti@unipv.it (B.C.); lauretta.maggi@unipv.it (L.M.); 2 Department of Otolaryngology, Fondazione IRCCS Policlinico San Matteo, 27100 Pavia, Italy; 3 Department of Chemistry, Physical-Chemistry Section, University of Pavia, Via Taramelli 16, 27100 Pavia, Italy; giovanna.bruni@unipv.it

**Keywords:** nanofibers, sustained release, fast release, tablets, polymer, poorly soluble drug, formulation, release kinetics

## Abstract

A challenge in the pharmaceutical sector is the development of controlled release dosage forms for oral administration of poorly soluble drugs, in particular, drugs characterized by pH-dependent solubility through the gastrointestinal tract, which itself shows wide variability in terms of environmental pHs. The best approach is to increase the dissolution rate of the drugs at the different pHs and only then modify its release behavior from the pharmaceutical form. This work aims to demonstrate the ability of properly designed polymeric nanofibers in enhancing the release rate of model drugs with different pH-dependent solubility in the different physiological pHs of the gastrointestinal tract. Polymeric nanofibers loaded with meloxicam and carvedilol were prepared using the electrospinning technique and were then included in properly designed tablet formulations to obtain fast or sustained release dosage forms. The nanofibers and the tablets were characterized for their morphological, physico-chemical and dissolution properties. The tablets are able to deliver the dose according to the expected release behavior, and zero-order, first-order, Higuchi, Korsmeyer–Peppas and Hixon–Crowell kinetics models were used to analyze the prevailing release mechanism of the tablets. This study shows that the electrospun fibers can be advantageously included in oral dosage forms to improve their release performances.

## 1. Introduction

Immediate release dosage forms are the traditional drug delivery systems. In the case of gastrolesive drugs, repeated dosing of the drug might lead to the risk of the burst effect and/or gastric mucosal damage including bleeding, ulceration and perforation [1]. For this reason, is advisable to have medicines capable to prevent releasing the active acidic conditions of the stomach, and to allow its release only in the intestinal environment. Furthermore, for some chronic therapies, a medicinal product capable of maintaining a near constant or uniform blood level could be more advantageous in clinic therapy [2].

Developing a controlled release system for very soluble drugs [3] and poorly soluble drugs [4,5] is very challenging. It is especially difficult to modulate the release of drugs characterized by low solubility and pH-dependent solubility through the gastrointestinal tract, which itself shows wide variability in terms of environmental pH. Controlled release delivery may have significant advantages for example by reducing the frequency of administration with consequent compliance of the patient, fewer side effects or improving the drug bioavailability and site specificity [6].

The best approach is to increase the dissolution rate of the poorly soluble drug at the different pHs (so that the solubility could be no longer pH-dependent) and only then to control its release properties from the pharmaceutical form. Many approaches have been used to make the drug more available in dissolution, such as the preparation of co-crystals [7,8], hybrid compounds [9,10,11], amorphous solid dispersions [12] drug-cyclodextrin complex [13] and supercritical carbon dioxide-based techniques [14,15].

Recently, electrospinning (ES) has demonstrated to be an attractive strategy to increase the release of poorly soluble drugs [16,17], ranging from antibiotics and anticancer agents to macromolecules such as proteins and DNA [18]. The improvement of the dissolution rate and/or solubility of the loaded drugs are achieved by the high surface-to-volume ratio of the electrospun nanofibers, the potential amorphization of the active and enhancement of its wettability. Moreover, the main feature of this technique is its ability to include a wide variety of drugs into polymeric carriers [19] to obtain loaded fibers able to enhance the drug dissolution rate and eventually their bioavailability. The production of electrospun fibers for drug delivery applications starts with polymer dissolution in a suitable solvent. Then, the drug is mixed into the polymeric solution, creating a homogeneous solution or a suspension (depending on drug’s solubility in the polymer solvent). This mixture is electrospun to produce dried nanofiber composed of a solid in which the drug is finely dispersed, up to molecular stage, in the polymer [20]. Polymer selection is a very critical decision for the development of a successful drug delivery system, according to their intrinsic properties such as water-solubility, swelling, surfactant and disaggregating properties that can be used to modulate drug release rate (faster or sustained) [21,22]. There are several biocompatible and biodegradable biomaterials, including bovine serum albumin (BSA) [23], gelatin [24,25,26], zein [27,28,29,30], polycaprolactone (PCL), polylactic acid (PLA) [31] and chitosan [32,33] used for oral drug delivery systems. The excipient widely used in oral sustained drug release devices is hydroxyl-propyl-methyl-cellulose (HPMC) [34,35]. HPMC is widely used in pharmaceutical research, development and manufacturing as it is available in numerous viscosity grades and molecular weights, providing great variability of its physico-chemical properties. Additionally, both the FDA and the EMA regulatory agencies classify HPMC as a safe pharmaceutical excipient (generally recognized as safe, GRAS) [36,37].

Hydroxypropylmethyl-cellulose acetate succinate (HPMC-AS) is a new derivative of HPMC obtained in different grades, namely HPMC-AS 716 and HPMC-AS 912 (Dow Chemical Company, Midland, MI, USA), that differ in the degree of acetyl- and succinyl- substitution. The use of one product rather than another can influence and modulate the optimization of desired drug formulation. HPMCAS, compared to HPMC, improves the physical stability of the solid systems by inhibiting drug re-crystallization [38], and due to its high swellability, has a significant effect in promoting the release kinetics of the incorporated drug. The combination of different grades of HPMC-AS and HPMC for the development of fibers could improve the release of poorly soluble drugs. Moreover, compared to other biomaterials cited above, zein, PCL and PLA are water insoluble and thus cannot improve the drug solubility for oral administration, whereas natural-based materials such as BSA, gelatin and chitosan, although soluble, are not suitable to enhance the drug solubility.

The work aimed to produce HPMC-AS electrospun nanofibers to demonstrate the ability of the electrospun system in increasing the release rate of two model drugs having different pH-dependent solubility in different physiological pH conditions: meloxicam (solubility 4.4 × 10^−3^ mg/mL in water at 25 °C; pKa: 4.08 [19]) and carvedilol (solubility 1.1·10^−2^ mg/mL in water at 37 °C; pKa: 7.8 [39]).

Additionally, to develop dosage forms with tailor-made release performances capable of ensuring the best therapy, the drug-loaded electrospun nanofibers were included in properly designed formulations to prepare tablets with immediate release or sustained release characteristics. The nanofibers and the tablets developed were characterized for their morphological (Scanning Electron Microscopy), physico-chemical (Differential scanning calorimetry and X-ray Powder Diffraction) and technological properties (contact angle and in vitro dissolution tests).

The dissolution performances of the dosage forms were tested in vitro in different media simulating the physiological conditions of gastrointestinal tract. Moreover, mathematical models (zero-order, first-order, Higuchi, Korsmeyer–Peppas and Hixson–Crowell) were used to evaluate the drug release kinetics from tablets.

## 2. Materials and Methods

Meloxicam (M) and Carvedilol (C) were kindly donated by AMSA S.p.A. (Milano, Italy) and Moehs (Barcelona, Spain) respectively. Hydroxypropylmethylcellulose Acetate Succinate, Affinisol^®^ 716G and Affinisol912G (to follow coded as HPMC-AS716 and HPMC-AS912) were supplied by the Dow Chemical Company (Bomlitz, Germany), hydroxypropyl methylcellulose: Methocel^®^ K100LV and MethocelK4M, (coded HPMC K100LV and HPMC K4M) were obtained through Colorcorn Limited (Darford, UK), microcrystalline cellulose NF(Avicel^®^ PH102) was supplied by FMC, (Philadelphia, PE, USA), cross-linked polyvinylpyrrolidone (Polyplasdone^®^ XL) was obtained through GAF, (New York, NY, USA), sodium starch glycolate (Primojel^®^) was supplied by ELKO (Milano, Italy), spray dried SD-Mannitol (Pearlitol^®^ 100) was supplied by Roquette (Lestrem, France) and magnesium stearate by Carlo Erba, (Milan, Italy).

Polymethacrylate-based copolymer (Eudragit^®^ L100-55) (Evonik, Essen, Germany) andTriethyl Citrate (TEC) (Rofarma Italia S.r.l., Gaggiano, Italy) were used as electrospun fibers coating.

Unless specified, all solvents and reagents were of analytical grade.

### 2.1. Polymer Electrospun Nanofibers Preparation

Different polymers were preliminarily screened to produce electrospun fibers to increase the drugs dissolution rate. Finally, two types of HMPC-AS, HPMC-AS716 and HPMC-AS912, were selected as drug carriers. Subsequently, HPMC-K100LV, a low-viscosity hydrocolloid was added to evaluate its ability to further enhance the drug dissolution rate. Electrospun fibers were obtained using Electrospinning set up NANON-01A equipped with a dehumidifier (MEEC instruments, MP, Pioltello, Italy). The main instrument parameters set up were: voltage 28–30 kV, flow-rate 0.4–0.6 mL/h, needle-collector distance 14 cm and needle size 18 G—22 G—25 G, and they were evaluated to obtain a homogeneous and continuous jet able to produce optimal nanofibers. Electrospinning process parameters were set up to obtain placebo electrospun fibers. An ethanol (EtOH) and methylene chloride (MC) blend 1:1 (*v*/*v*) ratio was used as the polymers solvent.

The dried fibers were deposited on an aluminum foil placed onto a flat plate metal collector. The temperature and humidity were set at 30 °C and 40% U.R., respectively, and they were constantly monitored to improve the evaporation of the solvent. The manufactured nanofibrous matrices were stored in a vacuum chamber for two days to remove solvent residual. The optimized electrospinning process parameters were used to manufacture nanofibrous electrospun matrices loaded with the two selected model drugs, meloxicam (M) and carvedilol (C), as follows.

M was uniformly suspended in the polymer solution because it is not soluble in the Ethanol (EtOH) and Methylene Chloride (MC) blend, whereas C was dissolved in the polymer solvent mixture. Therefore, a suspension was electrospun in the case of M, whereas a polymer solution containing C was electrospun.

Fibers loaded with M and C were coded with the acronyms Mfb and Cfb, respectively. A progressive number was then added to the acronyms to identify the composition of the electrospun polymeric solutions. In the results section, the composition and the electrospinning process parameters of the fibers prepared with HPMC-AS716, HPMC-AS912, HPMC K100LV and selected drugs are reported (Table 1).

### 2.2. Formulations and Tablets Preparation

Drug-loaded electrospun nanofibers that showed in vitro-enhanced drug dissolution rates were selected to prepare tablet formulations for fast and sustained release of the drugs. The loaded nanofibers were ground in a mortar. The proper amount of nanofibers was mixed with the excipients reported in tab 1in a Turbula apparatus (Turbula T2A; Bachofen, Basel, Switzerland) for 10 min.

The fast releasing tablets contained sodium starch glycolate or cross-linked polyvinylpyrrolidone as disintegrants and microcrystalline cellulose as a bulking agent. Instead, for sustained formulations, high molecular weight HPMC (Methocel K4M) was used to produce the matrix swelling and mannitol was used as the bulking agent (Table 2). The formulations were reported in the text, adding the letter c to the fiber acronyms Mfb4 or Cfb4. A progressive number was then added to the acronyms to identify each formulation.

The tablets were prepared using a single punch tablet machine (Korsch EK0; Korsch AG, Berlin, Germany) equipped with 5-6-8 mm in diameter round punches at 35 kN. The tablets are coded below with the letter -t added to the formulation code (i.e., Mfb4c1t).

Due to the meloxicam gastrolesivity [1], we explored a further application of the electrospinning process by fiber-coating M tablets with a solution of 20% *w*/*v* of polymethacrylate-based copolymer (Eudragit L100-55) in EtOH:MC 1:1 (*v*/*v*) ratio with and 2% *w*/*v* of triethyl-citrate (TEC) as a plasticizer. This kind of polymethacrylate-based copolymer dissolves only above a pH of 5.5, preventing M release in the gastric environment. A fiber-coating amount was evaluated by gravimetric analysis by weighing the tablets before and after coating. The letter r is added to the name of the tablet formulation to identify the coated tablets, i.e., Mfb4c1tr.

### 2.3. Morphological Characterization: Scanning Electron Microscopy (SEM)

A Zeiss EVO MA10 (Carl Zeiss, Oberkochen, Germany) was used to observe the morphology of the fibers. The samples were gold-sputter coated under argon to render them electrically conductive prior to the analysis.

The images obtained by SEM were processed using the ImageJ program (open-source image processing program designed for scientific multidimensional images—National Institutes of Health, Bethesda, MD, USA) and DiameterJ (online source, DiameterJ v1.018 ImageJ software, USA) plugin to achieve the data of fiber size [40].

### 2.4. Differential Scanning Calorimetry (DSC) and X-ray Powder Diffraction (XRPD)

The DSC measurements were performed by a DSC Q2000 apparatus interfaced with a TA 5000 data station (TA Instruments, New Castle, DE, USA) in open standard aluminum pans under nitrogen flow (45 mL·min^−1^) at 10 K·min^−1^. The DSC instrument was calibrated using ultrapure (99.999%) indium (melting point = 156.6 °C; ΔH = 28.54 J·g^−1^) as standard. 

XRPD measurements were performed by a D5005 Bruker diffractometer (Malvern Panalytical, Karlsruhe, Germany) (CuKα radiation, λ (Kα1) = 1.54056 Å; voltage of 40 kV and current of 40 mA) equipped with a θ-θ vertical goniometer, Ni filter, monochromator and scintillator counter. The patterns were recorded at room temperature in step scan mode (step size: 0.02°, counting time: 3 s per step) in the 3 < 2 θ° < 30 angular range.

### 2.5. Dissolution Tests

The USP paddle method (Erweka DT-D6, Erweka GmbH, Dusseldorf, Germany) was used to evaluate the drug dissolution profiles from the fibers and tablets, at 37.0 ± 0.5 °C (n = three replicates).

The samples containing C were tested in 900 mL of pH 6.8 phosphate buffer at 50 rpm. This buffer is chosen because C is much more soluble at lower pHs and it dissolves easily (data not shown), whereas it is poorly soluble at pH 6.8. Thus, only in these conditions it is possible to evaluate possible improvements in the in vitro performances of this drug.

The samples containing M were tested using two different pH-change methods to evaluate the gastro-resistance of the coated tablets in acid conditions, and then the coating dissolution and drug release at pH 7.2 buffer that simulates intestinal conditions, at 75 rpm. In the first pH-change method, the samples were placed in 750 mL of 0.1 N hydrochloric solution, pH 1 (simulating gastric fluid), for the first 2 h and then 250 mL of Na_3_PO_4_ 0.2 M was added to the medium to reach pH 7.2 (Extended-release dosage forms. Dissolution Method [41]). The second method was modified to simulate the fed state: the samples were placed in 950 mL of pH 4.5 phosphate buffer for 4 h [42] and then 10 mL of NaOH 2 N was added to reach pH 7.2. The drug concentrations were measured by a UV-Vis spectrophotometer (Lambda 25, Perkin-Elmer, Monza, Italy) on filtered portions of the dissolution fluid, at 362 nm for M and 286 nm for Cand, respectively. The data were processed by a suitable software (Winlab V6 software, Perkin-Elmer, Monza, Italy).

All the dissolution media were prepared according to the USP reagents Section [43].

Finally, to highlight the differences found in the dissolution profiles of the different formulations, the time to reach 90% of the drug dissolved (td 90%) was calculated.

### 2.6. Contact Angle

Contact Angle Meter DMe-211Plus (NTG Nuova Tecnogalenica, Cernusco, Italy) was used to compare the wettability of the actives and the loaded nanofibers: a 10 µL drop of different fluids: a hydrochloric 0.1 N solution of pH = 1.0 or pH 4.5, pH 6.8 and pH 7.2 phosphate buffers was extruded from the needle and dropped onto the solid surface of the samples. The images of the drop in contact with the samples were acquired in progressive times (from *t* = 0 up to 60 s) and suitable software was provided to measure the contact angle. Three replicates were performed for each sample.

### 2.7. Release Kinetics

Zero-order, first-order, Higuchi, Korsmeyer–Peppas and Hixon–Crowell kinetics models were used to define the drug release mechanism from the tablets proposed [44].

A Zero-order kinetics is obtained when drug release rate is quite constant during the entire liberation process from pharmaceutical forms that show no disaggregation and slow drug dissolution rates [45]. The slope of the graph obtained plotted the percentages of drug dissolved against time (hours), giving the zero-order rate constant (1)

C_0_ − C_t_ = K_0_t (1)

C_t_ is the amount of drug dissolved at time t, whereas C_0_ is the initial concentration of active at time *t* = 0 and K_0_ is the zero-order rate constant.

When the release rate is directly proportional to drug concentration, a first-order kinetics model is used to describe the drug release mechanism. This model can be used to define the initial dissolution stage of a poorly soluble drug from a hydrophilic system. Log of percentage of the remaining drug in the tablet was plotted against time (hours) and the slope of the plot provides the first-order rate constant [46] (2)

log C = log C_0_ − K_1t_/2.303 (2)

Value K_1t_ is the first-order rate equation expressed in time or per hour and C_0_ is the initial concentration of the active, whereas C is the percent of active remaining in the tablet at time t.

The Higuchi model is represented by Equation (3) and expresses the drug release from a system that involves both dissolution and diffusion mechanisms [47]. To obtain the Higuchi model plot, the percentage of dissolved drug against the square root of time (t^1/2^) was used.

(3) $$Q=A\sqrt{D\;\left(2C_{0}-C_{s}\right)C_{s}t}$$

Value Q indicates the cumulative amount of active released in time t per unit area and C_0_ is the initial active concentration, whereas C_S_ is the drug solubility in the matrix and D is the diffusion coefficient of the drug molecule in the matrix. This model describes a diffusion controlled release mechanism. 

The highest correlation coefficient (R^2^) derived from kinetics models will be used to describe the prevailing drug release mechanism from the different tablet formulations.

If is confirmed that the drug release is diffusion controlled by the Higuchi equation, it is possible to plot the dissolution data using the Korsmeyer–Peppas model (4) to describe the type of drug release mechanisms from tablets.

M_t_/M_∞_ = K_kp_t^n^ (4)

M_t_/M_∞_ indicates the fraction of active released at time t, n is the diffusional exponent or drug release exponent and K_kp_ is the Korsmeyer release rate constant [48]. The log of the percentage active dissolved as a function of log time is plotted. This equation can be used to analyze the first 70% of a release curve, regardless of the geometric shape [3]. To describe the release mechanisms for cylindrical shaped matrices, an n (release exponent) value is used. When n > 0.5 drug transport mechanism follows Fickian diffusion, 0.5 < n < 0.89 describes a non-Fickian transport, n = 0.89 expresses a Case II transport and n values higher than 0.89 refer to super case II transport [49].

Finally, the Hixson–Crowell kinetics model defines the release process from tablets which involve a change in surface area and diameter but where the geometrical shape keeps constant [50].

(5) $$W_{0}^{1/3}-W_{t}^{\frac{1}{3}}=K_{\mathrm{HC}}t$$

W_0_ indicates the initial amount of active, whereas Wt is the remaining amount of active in the pharmaceutical form at time *t*; K_HC_ is the Hixson–Crowell constant describing surface to volume relation. Data were plotted as the cube root of drug percentage remaining in the tablet versus time. Using this model, if the correlation coefficient (R^2^) is higher than 0.9, it is possible to interpret that drug release is influenced by a change in surface area during the dissolution process.

## 3. Results

### 3.1. Polymeric Electrospun Nanofibers Preparation

The spinneret-collector distance was fixed at 14 cm to allow solvent evaporation and collect dry fibers. A 22 Gauge (G) needle (inner diameter: 0.759 mm) was chosen to obtain nanofibers. A smaller needle (25 G) was tried but it was often blocked by the polymer solution, whereas when using a needle with a larger diameter (18 G), the solution dripped and it was not possible to obtain a continuous jet. Environmental parameters, such as temperature and relative humidity, were kept constant at 30 ± 3 °C and 40 ± 5%, respectively.

Fiber-coating with polymethacrylate-based copolymer was performed using 30 kV and 1 mL/h flow-rate. A 15 min fibers coating was performed for each tablet surface and gastro-resistance was tested during the dissolution test. Fiber coating was homogeneous for each tablet covered and caused a weight increase of 3 ± 0.3%.

### 3.2. SEM Measurements

**Figure 1:**
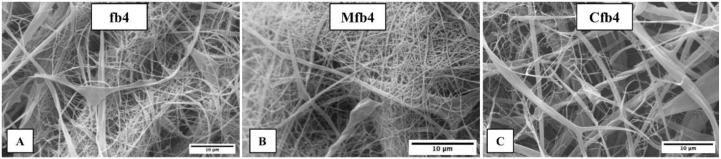
SEM photographs of (**A**) placebo HPMC-AS912 and HPMC K100LV electrospun fibers (fb4); (**B**) meloxicam loaded fb4 electrospun fibers (Mfb4); (**C**) carvedilol loaded fb4 electrospun fibers (Cfb4).

The fibers dimensions were in the sub-micrometric range (Figure 1). In some portions, flat and fused fibers are seen, probably due to the deposition of fibers still being wet during the electrospinning process. The addition of drug did not significantly change the average fiber size compared to the placebo. Drug particles are not visible on the fibers surface.

The SEM images obtained were processed by ImageJ software to obtain values of the fibers diameter. Placebo electrospun fibers (fb4) showed a diameter of 0.41 ± 0.17 µm. In some portions, flat and fused fibers are seen, probably due to the deposition of fibers still being wet during the electrospun process. M-loaded fibers (Mfb4) exhibited a fiber diameter of 0.39 ± 0.08 µm, whereas C-loaded fibers (Cfb4) were 0.44 ± 0.24 µm. The addition of the drug to the fibers did not significantly change the average fiber diameter compared to the placebo ones. Concerning the intra-fibers porosity, SEM images showed for all electrospun fibers obtained, smooth and non-porous structures can be seen.

### 3.3. DSC and XRPD Measurements

**Figure 2:**
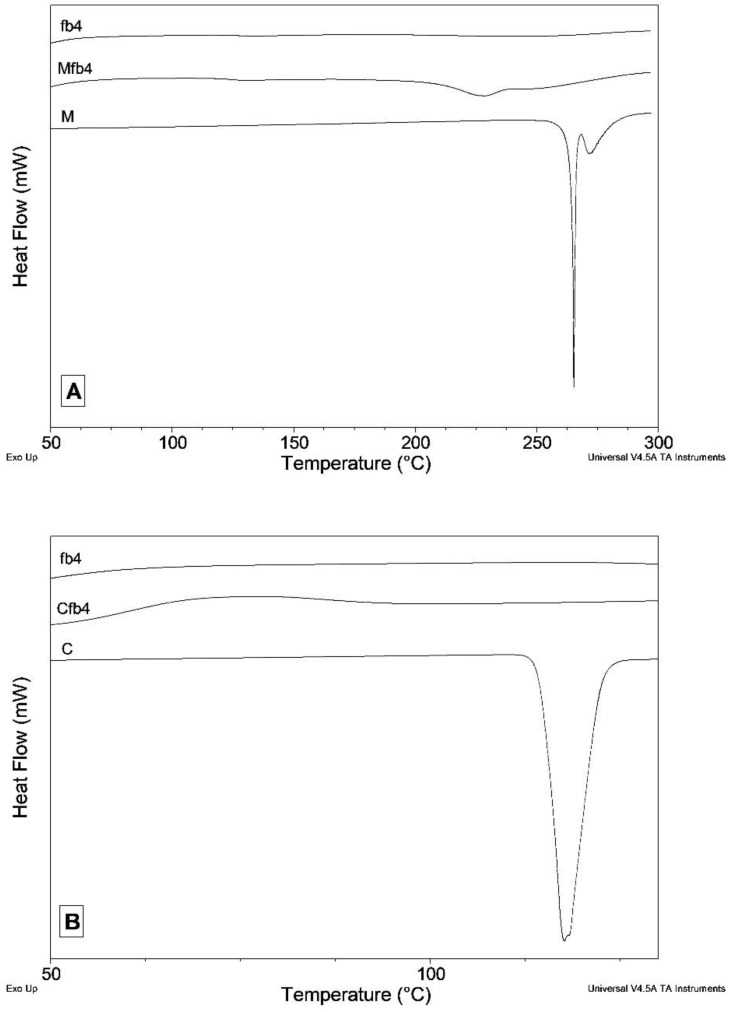
DSC curves of meloxicam, fb4 and Mfb4 (**A**) and carvedilol, fb4 and Cfb4 (**B**).

In the meloxicam DSC trace, a double endothermic effect is observed, attributable to the processes of melting and decomposition that occur almost simultaneously (Figure 2A).

In the DSC trace of the fibers (Figure 2A), the endothermic effect falls at a significantly lower temperature (Tonset = 206 °C instead of 265 °C; the peak opens about 80 °C earlier than the pure drug) and is very broad. For both samples, there is an exothermic drift of the baseline after the peak, due to decomposition process, which does not allow a reliable integration of the peak. An attempt was also made to carry out the integration. The enthalpy change measured for the MFb4 sample is approximately 22% of the value measured for pure meloxicam. Therefore, there is good correspondence with the value expected in the hypothesis that all meloxicam loaded in the fibers is in the crystalline phase. The fact that endothermic effect in the fibers DSC curve is anticipated compared to that measured for pure meloxicam suggests that the particles of M loaded into the fibers have a much smaller size than the original drug powder. This leads to a lowering of the melting/decomposition temperature.

**Figure 3:**
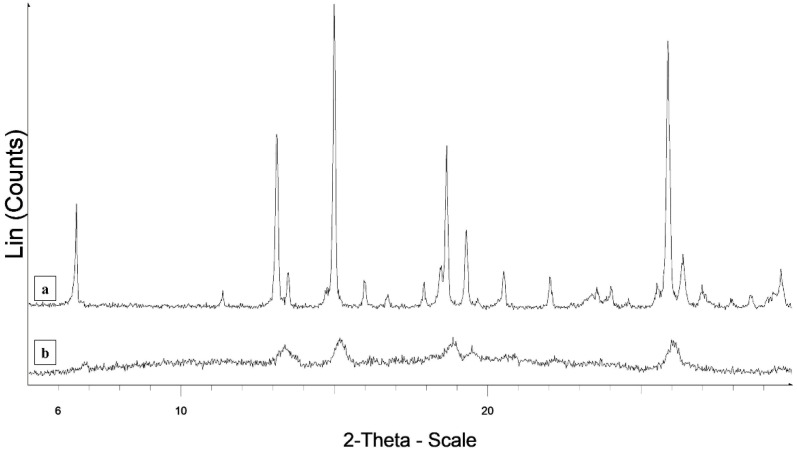
XRPD patterns of meloxicam (a) and MFb4 (b).

The X-ray patterns of pure M exhibit diffraction peaks typical of a crystalline powder (Figure 3). In the XRPD pattern collected on the fibers on the hump due to the amorphous material constituting the fibers, diffraction effects are present in the same angular positions corresponding to the most intense diffraction peaks of pure meloxicam. However, these effects are very broadened, as expected in the case of particle size reduction even to a nanometer scale. The XRPD diffraction measurements therefore fully confirm the deductions drawn from the DSC measurements: the M particles loaded on the fibers are still crystalline, but particle size is significantly different.

C melts at 114.9 °C with an enthalpy variation of 117.5 J·g-1 (Figure 2B). The melting peak is no longer visible in the DSC curve of the CFb4 fibers (Figure 2B), indicating that the C loaded in the fibers is in the amorphous phase.

**Figure 4:**
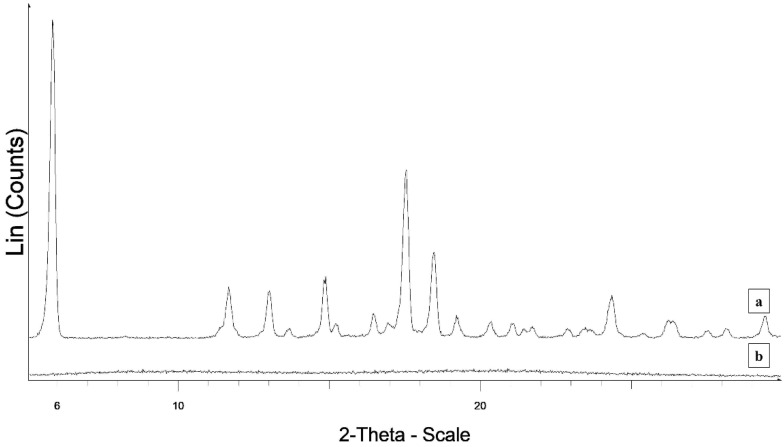
XRPD patterns of carvedilol (a) and CFb4 (b).

The XRPD pattern of C shows peaks typical of a crystalline powder, whereas that of the fibers is typical of an amorphous sample (Figure 4).

The DSC and XRPD results agree on suggesting that the drug loaded in the fibers is in the amorphous phase.

### 3.4. Dissolution Tests and Contact Angle

**Figure 5:**
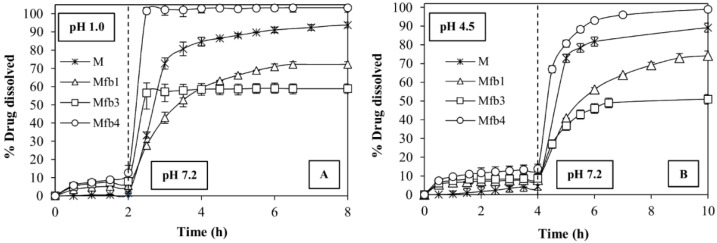
Dissolution profiles of M, and drug-loaded fibers obtained by HPMC-AS 716 (Mfb1), HPMC-AS912 (Mfb3), and by combining HPMC-AS912 and HPMC K100LV (Mfb4) in two different pH change methods: pH 1.0 for 2 h and then pH 7.2 (**A**) and pH 4.5 for 4 h and then pH 7.2 (**B**). All samples contain a dose of 7.5 mg of M.

The fibers made of HPMC-AS716 (Mfb1) and HPMC-AS912 (Mfb3) do not show any improvement of M dissolution rate in the second phase of the two pH-change methods i.e., in pH 7.2 buffer compared to free M (Figure 5A,B), whereas the combination of HPMC-AS912 and HPMC K100LV (Mfb4) shows a remarkable increase of the dissolution rate of this drug; in fact, Mfb4 releases the entire dose in the pH 7.2 buffer in a very short time. At the same time, however, Mfb4 releases about 10% of the dose in the first phase at pH 1.0 and pH 4.5.

**Figure 6:**
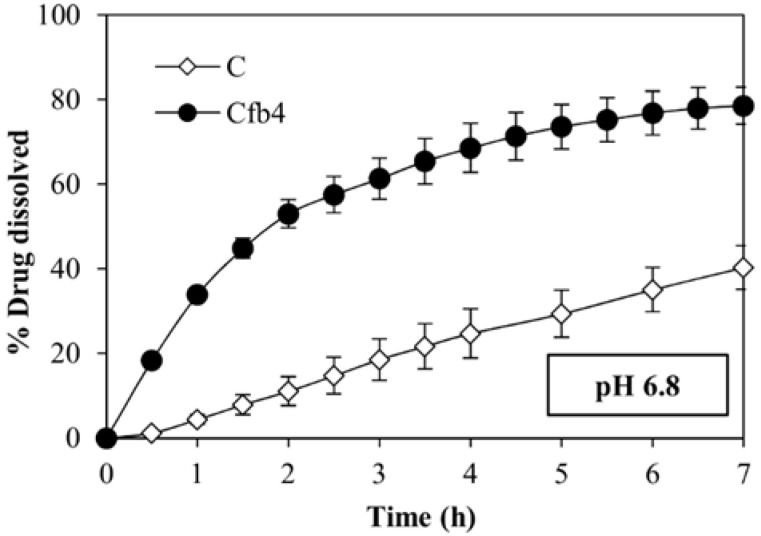
Dissolution profiles of carvedilol alone (C) and Cfb4 drug-loaded fibers in pH 6.8 buffer. All samples contain a dose of 6.25 mg of carvedilol.

The same polymeric combination (HPMC-AS912 + HPMC K100LV) was able to enhance the dissolution rate of C and Cfb4 in pH 6.8 buffer compared to the drug alone, which is much less soluble at this pH (Figure 6).

**Figure 7:**
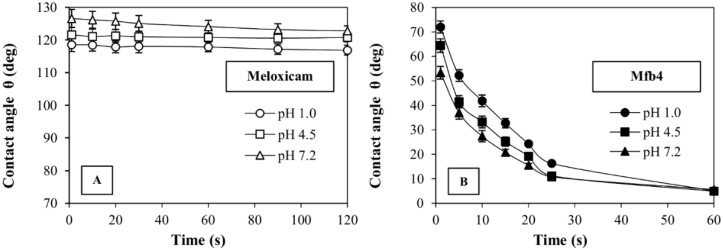
Contact angle values (θ), as a function of time, for meloxicam (**A**) and Mfb4 (**B**) in the different media considered (pH 1.0, pH 4.5 and pH 7.2).

**Figure 8:**
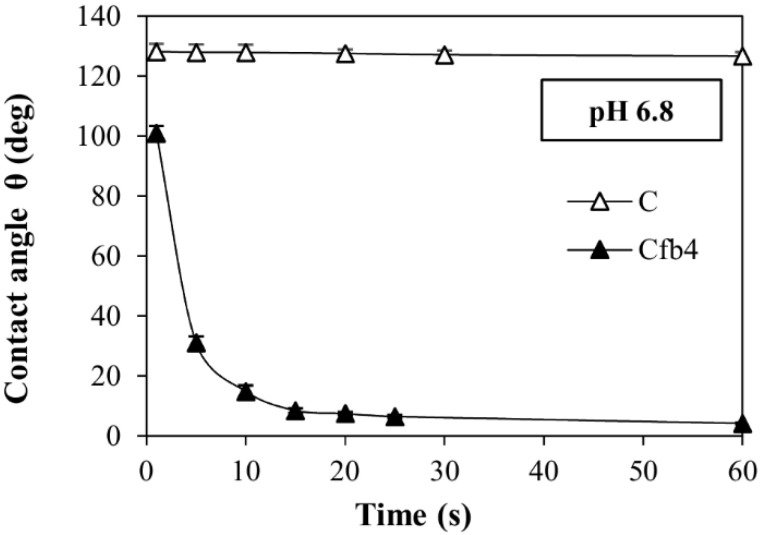
Contact angle values (θ), as a function of time, for carvedilol and Cfb4 in the pH 6.8 buffer.

The fibers produced using the combination of these two polymers also show a notable increase in wettability. Their contact angle, measured in the different fluids, is compared to the drugs in powder in Figure 7B (Mfb4) and Figure 8 (Cfb4), and shows significant lowering in all cases. A contact angle of Mfb4 is θ < 10° after 60 s in the three buffers tested was noted; Cfb4 reaches values θ < 10° already after 15 s of contact with the pH 6.8 buffer. These contact angle values account for the remarkable increase of the drug dissolution rate. On the contrary, the contact angle of pure M and C remains constant, θ > 110°, for long times, demonstrating their prevailing hydrophobic characteristics (Figure 7A and Figure 8).

**Figure 9:**
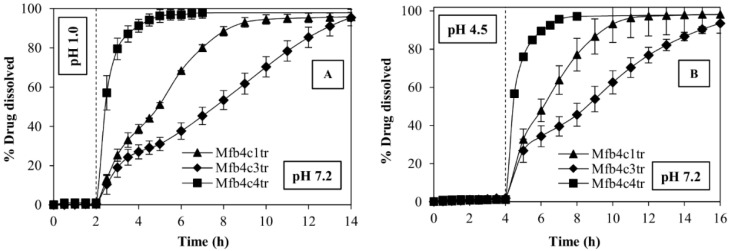
In vitro dissolution profiles of fast release formulation (Mfb4c4tr) and of two sustained release formulations (Mfb4c1tr and Mfb4c3tr) in the two different pH change methods: pH 1.0 for 2 h and then pH 7.2 (**A**), pH 4.5 for 4 h and then pH 7.2 (**B**). All samples contain a dose of 7.5 mg of M.

The combination of HPMC-AS 912 and HPMC K100LV (fb4) allowed for increasing and normalizing the in vitro performances of the two model drugs, M in pH 7.2 and C in pH 6.8 buffers. Thus, Mfb4 and Cfb4 were formulated to obtain tablets with different drug release behaviors (fast or sustained) (Figure 9).

Mfb4c1tr and Mfb4c3tr formulations allow achieving M sustained release. The dose is released at different rates because the percentage of HPMC of high molecular weight used (Methocel K4M) modulates the swelling/release properties in the two formulations (Table 2). Instead, the use of super-disintegrating agents in Mfb4c4tr formulation allows for achieving a fast drug release. Results are supported by the td 90% values (Table 3). Mfb4c3tr needed 5 h more than Mfb4c1tr to reach td 90% in pH 7.2 (after the pH change), and this lag time is the same in both the tests performed (simulating fast and fed conditions), whereas Mfb4c4tr achieves td 90% already within 2 h.

The tablet formulations were fiber-coated with polymethacrylate-based copolymer (Eudragit L100-55) to prevent the release of the drug in acidic conditions, and in fact, the M release was completely prevented in the first phases of the pH change methods.

**Figure 10:**
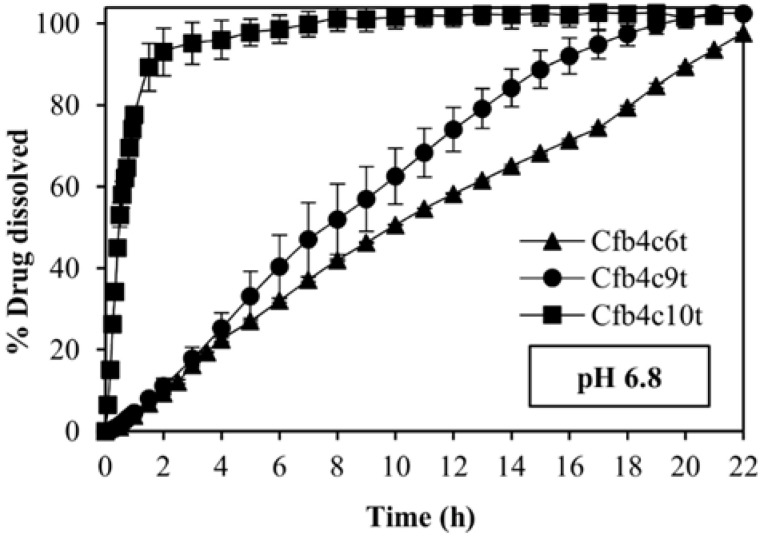
Dissolution profiles of the fast release formulation, Cfb4c10t and two sustained release formulations, Cfb4c6t and Cfb4c9t in the pH 6.8 buffer. All samples contain a dose of 6.25 mg of carvedilol.

Cfb4c6t and Cfb4c9t allow reaching a sustained release of C, showing a td 90% of 20 h and 15 h respectively. Instead, Cfb4c10t allows achieving a fast drug release, reaching a td 90% in 1.5 h. (Figure 10).

### 3.5. Release Kinetics

Kinetic models evaluated for sustained release formulations of M (Mfb4c1tr and Mfb4c3tr) highlighted that the tablets follow preferentially the Higuchi model, which implies that the drug was released mainly through a diffusion process (Table 4).

Korsmeyer–Peppas power law equation gives more in-depth information: an n value higher than 0.89 implies that the drug release from the tablets follows super case II transport and that drug release is swelling controlled. Moreover, when the correlation coefficient of the Hixson–Crowell equation is high (R^2^ > 0.9), a progressive change in the matrix surface area during the process of dissolution has a significant effect on drug release. In this case, a combination of swelling and erosion mechanisms of the tablets as a function of time can be expected.

Kinetics models evaluated for C tablets highlighted that the systems follow predominantly the zero-order model (Table 4). High values of the Hixson–Crowell equation highlighted that the erosion process during swelling has a significant effect on drug release.

## 4. Discussion

The electrospinning technique has allowed obtaining HPMCAS and HPMC-K100LV fibers (fb4) of nanometric dimensions, guaranteeing a high surface area exposed to fluids.

The fibers obtained proved valid support for the delivery of two drugs, M and C, which have different pH-dependent solubilities. The fibers loaded with M (Mfb4) showed from DSC and XRPD analysis that the loaded drug remained in crystalline form, but its particle size was reduced compared to free M. The reduction of the particle size to a nanometric scale allows for obtaining a larger surface area exposed to the dissolution medium. On the contrary, C loaded in fb4fibers becomes an amorphous form. This different behavior may also be due, in addition to different crystallization properties of the two drugs, to their different solubility in the solvents used for the electrospinning process, ethanol and methylene chloride. In the case of M, a homogeneous suspension is obtained, whereas C is completely soluble in the solvent mixture.

The combination of HPMC-AS912 and HPMC K100LV in the electrospun Mfb4 and Cfb4 fibers shows an increase in the dissolution rate of both drugs compared to the free drugs in the fluids considered. This effect could be due to the hydrophilic properties of the carrier polymers that enhance the wettability of the actives. Furthermore, the surface area increases, due to the sub-micrometric dimensions of the fibers, and, in the case of C, to its amorphization as well.

Once the dissolution performances of the drugs are increased and normalized in the different pHs, different formulations of the fibers to obtain a fast release or sustained release tablets of the model drugs can be designed.

In the case of M that may show gastrolesive effects, a coating can even be applied using the electrospinning process. A layer of polymethacrylate fibers placed on the tablets surfaces has shown to prevent M release in an acidic environment at pH 1.0 for at least 2 h and at pH 4.5 for 4 h (conditions to simulate fasted and fed states).

Finally, the release kinetics were evaluated on the dissolution data of M in pH 7.2 and of C in pH 6.8. The sustained release formulations of the two actives show different release mechanisms: Higuchi model for M formulations and zero-order kinetics for C formulations. However, both sustained delivery systems release the drug at a constant rate, and this could maintain drug concentrations within the therapeutic window for an extended period of time. This dissolution behavior can be successfully used to limit side effects, reduce the frequency of administration and improve patient compliance.

## 5. Conclusions

The results obtained permit to conclude that through the electrospinning process applied to HPMC-AS and HPMC K100LV, it is possible to obtain sub-micrometric fibers capable of influencing the dissolution rate of drugs with low and pH-dependent solubility, at which point the fibers can be properly formulated in fast or sustained release tablets to satisfy therapeutic needs. In these terms, electrospun sub-micrometric fibers act as an intermediate of production, such as granulation, with interesting properties that can be tailored either according to their composition or fibers structure. Moreover, the polymethacrylate-based copolymer electrospun fibers showed the ability to act as a gastro-resistant barrier to reduce the release of gastro-damaging drugs, such as meloxicam, at pH values typical of the stomach.

This work is a proof of a concept to validate the ability of tableted methylcellulose-based nanofibers to control and increase the release of different kinds of drugs used as models. The proposed platform could be used with other types of drugs or with nanofibers produced with other manufacturing techniques to obtain further evidence of the capacity to modulate the drug release rate in different physiologic-like conditions.

## Figures and Tables

**Table 1:** Composition of the electrospun polymeric solutions and process parameters set up.

Composition of Electrospun Polymer Solutions						Electrospinning Process Parameters	
Fibers	HPMC-AS716 (%*w*/*v*)	HPMC-AS912 (%*w*/*v*)	HPMC K100LV (%*w*/*v*)	M (%*w*/*v*)	C (%*w*/*v*)	Voltage (kV)	Flow Rate (mL/h)
fb1	20.0	-	-	-	-	28	0.6
fb3	-	15.0	-	-	-	30	0.6
fb4	-	15.0	2.0	-	-	28	0.8
Mfb1	20.0	-	-	5.0	-	30	0.5
Mfb3	-	15.0	-	5.0	-	30	0.5
Mfb4	-	15.0	2.0	5.0	-	30	0.5
Cfb4		15.0	2.0	-	5.0	30	0.4

**Table 2:** Single unit composition of the fast release formulations / sustained release formulations containing fibers and diameter of the tablet obtained.

Ingredients	Fast Release Formulations		Sustained Release Formulations			
	Mfb4c6	Cfb4c10	Mfb4c1	Mfb4c3	Cfb4c6	Cfb4c9
Mfb4 (corresponding 7.5 mg of meloxicam)	33.0 mg	-	33.0 mg	33.0 mg	-	-
Cfb4 (corresponding 6.25 mg of carvedilol)	-	27.5 mg	-	-	27.5 mg	27.5 mg
Sodium starch glycolate	50.0 mg	-	-	-	-	
Microcrystalline cellulose	75.0 mg	100.0 mg	-	-	-	
Cross-linked polyvinylpyrrolidone	-	50.0 mg	-	-	-	
HPMC K4M	-	-	10.0 mg	30.0 mg	45.0 mg	33.0 mg
Mannitol	-	-	-	-	70.0 mg	70.0 mg
Magnesium stearate	1.0 mg	1.0 mg	1.0 mg	1.0 mg	1.0 mg	1.0 mg
**Total weight**	**159.0 mg**	**178.5 mg**	**44.0 mg**	**64.0 mg**	**143.5 mg**	**131.5 mg**
**Diameter of tablet**	8 mm	8 mm	5 mm	6 mm	8 mm	8 mm

**Table 3:** Time required for dissolution of 90% of M dose (td 90%) from the sustained release tablet formulations after the pH change.

Sample	td 90% After 2 h at pH 1.0	td 90% After 4 h at pH 4.5
**Fast release formulation (Mfb4c4tr)**	2 h	2 h
**Sustained release formulation 1 (Mfb4c1tr)**	6.5 h	5.5 h
**Sustained release formulation 2 (Mfb4c3tr)**	11 h	11 h

**Table 4:** Results of mathematical models of release kinetics (zero-order, first-order, Higuchi, Korsmeyer–Peppas and Hixson–Crowell) applied on M and C sustained release formulations.

Sustained Release Formulation	Zero-Order	First-Order		Higuchi	Korsmeyer–Peppas		Hixson–Crowell
	R^2^	R^2^	K_1_	R^2^	R^2^	n	R^2^
**MFb4c1tr** after 2 h in pH 1.0	0.9902	0.9628	−0.1547	**0.9945**	0.9505	1.7155	0.9866
**Mfb4c3tr** after 2 h in pH 1.0	0.9873	0.8986	−0.0966	**0.9889**	0.9454	1.1917	0.9593
**MFb4c1tr** after 4 h in pH 4.5	0.9860	0.9789	−0.1797	**0.9943**	0.9481	2.5252	0.9921
**Mfb4c3tr** after 4 h in pH 4.5	0.9879	0.9643	−0.0862	**0.9934**	0.9609	1.3467	0.9885
**Cfb4c6t**	**0.9929**	0.8764	−0.0334	0.9680	0.9351	2.4742	0.9586
**Cfb4c9t**	**0.9896**	0.9338	−0.0434	0.9664	0.9226	1.3374	0.9835

## Data Availability

The data presented in this study are available on request from the corresponding author.
